# A Chromosome-Level Genome Assembly and Annotation for the *Oecanthus rufescens* (Orthoptera: Oecanthidae)

**DOI:** 10.1093/gbe/evae145

**Published:** 2024-07-01

**Authors:** Xuan Jing, Hui-Yao Zhao, Yan-Na Zheng, Yi-Meng Nie, Li-Bin Ma, Yuan Huang

**Affiliations:** College of Life Sciences, Shaanxi Normal University, 710119 Xi’an, China; College of Life Sciences, Shaanxi Normal University, 710119 Xi’an, China; College of Life Sciences, Shaanxi Normal University, 710119 Xi’an, China; College of Life Sciences, Shaanxi Normal University, 710119 Xi’an, China; College of Life Sciences, Shaanxi Normal University, 710119 Xi’an, China; College of Life Sciences, Shaanxi Normal University, 710119 Xi’an, China

**Keywords:** crickets, genome assembly, gene annotation, repeat annotation

## Abstract

*Oecanthus* is a genus of cricket known for its distinctive chirping and distributed across major zoogeographical regions worldwide. This study focuses on *Oecanthus rufescens*, and conducts a comprehensive examination of its genome through genome sequencing technologies and bioinformatic analysis. A high-quality chromosome-level genome of *O. rufescens* was successfully obtained, revealing significant features of its genome structure. The genome size is 877.9 Mb, comprising ten pseudo-chromosomes and 70 other sequences, with a GC content of 41.38% and an N50 value of 157,110,771 bp, indicating a high level of continuity. BUSCO assessment results demonstrate that the genome's integrity and quality are high (of which 96.8% are single-copy and 1.6% are duplicated). Comprehensive genome annotation was also performed, identifying approximately 310 Mb of repetitive sequences, accounting for 35.3% of the total genome sequence, and discovering 15,481 tRNA genes, 4,082 rRNA genes, and 1,212 other noncoding genes. Furthermore, 15,031 protein-coding genes were identified, with BUSCO assessment results showing that 98.4% (of which 96.3% are single-copy and 1.6% are duplicated) of the genes were annotated.

**Significance**

The completion of the high-quality chromosomal-level whole-genome assembly of *Oecanthus rufescens* marks the establishment of the first reference genome within the Oecanthidae, order Orthoptera. These assembly data fill the gap in genetic data for this widely distributed cricket species, providing valuable resources and references for further exploration of the genetic functions and evolutionary processes of crickets.

## Introduction

The Grylloidea currently consists of around 6,300 extant species, making it the third-largest group within the Orthoptera (Cigliano et al. 2024). They are commonly found inhabiting tree leaves, grass patches, and shrubbery. The *Oecanthus* Serville, 1831, classified within the family Oecanthidae, Grylloidea, currently includes 78 species worldwide. *Oecanthus* are characterized by their slender bodies, thin and translucent wings, and coloration, typically yellow or green, commonly found in leaves, grasses, and shrubs. The *Oecanthus* are distributed widely across the world's major animal biomes, suggesting a long global evolutionary history and stable environmental adaptation capability. One of their distinctive features is that male crickets produce sound by friction of their wings, encompassing various chirping types such as calling, courtship, aggression, warning, and triumph (Wagner 1996; Hirtenlehner and Römer 2014). Although the morphology of this cricket group is relatively conserved, its chirping exhibits diversity, which enables researchers to distinguish cricket species based on their calls. Nevertheless, the majority of studies on chirping have concentrated on taxonomy, with little attention paid to the genetic level (Toms and Otte 1988; Collins and Schneider 2020; Collins et al. 2021). By mapping and analyzing these specific genes and regulatory networks, it is possible to gain a deeper understanding of the genetic control of chirping and the genetic mechanisms underlying its variability. Furthermore, it is possible to identify which genes are activated or repressed during specific chirping behaviors. This information facilitates the establishment of a direct link between behavioral phenotypes and molecular mechanisms, thereby further elucidating the genetic regulation of behavior. Consequently, a high-quality genome is the foundation and prerequisite for the aforementioned research.

Currently, the National Center for Biotechnology Information (NCBI) has publicly released genome data for six species within the Grylloidea, including five species from the Gryllidae: *Acheta domesticus* Linnaeus, 1758 (Gupta et al. 2020), *Gryllus bimaculatus* De Geer, 1773 and *Nudilla kohalensis* Otte, 1994 (Ylla et al. 2021), *Teleogryllus occipitalis* Serville, 1838 (Kataoka et al. 2020), and *Teleogryllus oceanicus* Le Guillou, 1841 (Kataoka et al. 2022). Additionally, two species from the Trigonidiidae are included: *Apteronemobius asahinai* Yamasaki, 1979 (Satoh et al. 2021), and *N. kohalensis* Otte, 1994 (Blankers et al. 2018). However, only the genome of *G. bimaculatus* has been fully sequenced at the chromosome level. This study aims to provide high-quality chromosome-level whole-genome data for the Oecanthidae. The research aims to elucidate the genomic structure and genetic diversity of *Oecanthus rufescens* to provide a new data foundation for further exploration of the evolutionary history, interspecies relationships, and biological characters of crickets.

## Results and Discussion

### Genome Size Estimation

The estimated genome size of *O. rufescens* is approximately 896.89 Mb (refer to supplementary fig. S1, Supplementary Material online), with a heterozygosity rate of about 2.36%, and the proportion of repetitive sequences standing at 42.14%. Using flow cytometry, the genome size of *O. rufescens* was identified as 1.08 Gb (refer to supplementary fig. S2, Supplementary Material online).

### Genome Assembly and Assessment

First, we aligned the obtained genome sequencing data against the NCBI Nucleotide Database. The results indicated that the data were not contaminated with exogenous DNA (refer to supplementary table S1 to S3, Supplementary Material online). After quality control, 45.69 Gb of high-quality data were acquired. The genome was preliminarily assembled to be 1,260,246,573 bp, consisting of 1,052 contigs. The longest contig was 23,011,083 bp, with an average length of 1,197,953 bp, and an N50 of 3,871,008 bp. Then, the sequencing data were used to remove duplication. This resulted in a draft genome of *O. rufescens* with a size of 877,827,037 bp, consisting of 403 contigs. The longest contig remained at 23,011,083 bp, with an increased average length of 2,178,231 bp, and an N50 of 4,538,698 bp (refer to Table 1).

**Table 1:** Summary statistics for the *O. rufescens* genome assembly and annotation

Assembly statistics	Value
Assembly length (bp)	877,902,837
# scaffolds	403
Scaffold N50 (bp)	2,178,231
Scaffold N90 (bp)	4,538,698
# contigs	1,052
Contig N50 (bp)	1,197,953
Contig N90 (bp)	3,871,008
GC content (%)	41.38
Hi-C anchored rate (%)	92.31
Gene annotation	
# protein-coding genes	15,031
# genes with functional information	12,823
# tRNA genes	15,806
# putative ncRNA genes	42,218
BUSCO analysis	
Complete (C)	98.4%
Single-copy (S)	96.8%
Duplicated (D)	1.6%
Fragmented (F)	0.3%
Missing (M)	0.3%

**Fig. 1.**
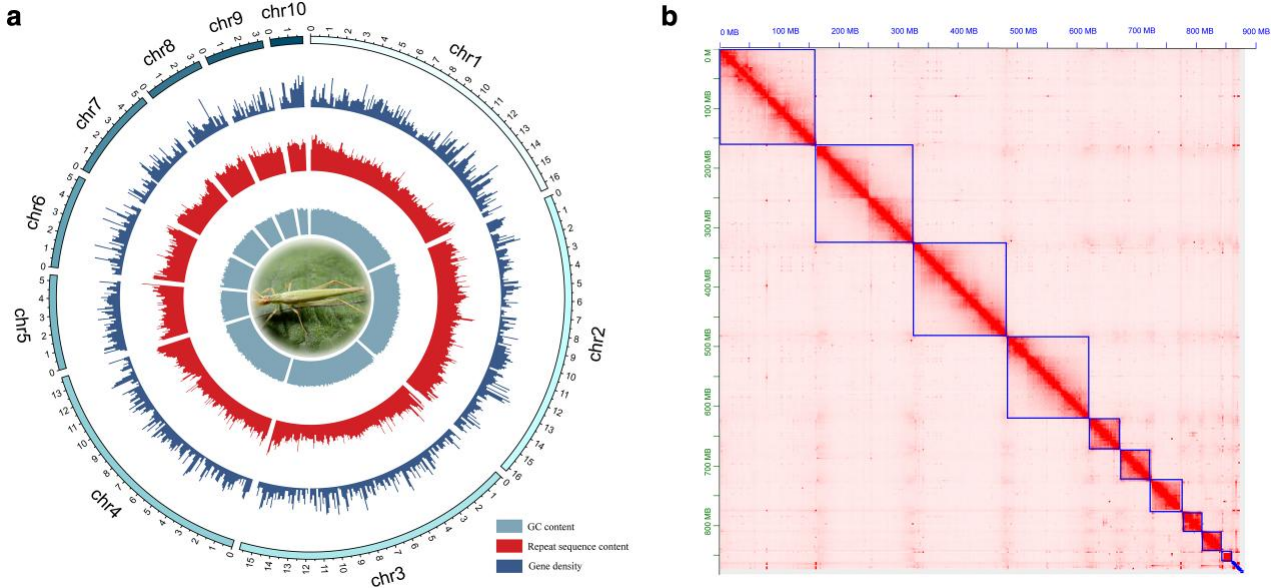
(a) A Circo map depicting the characters of the *O. rufescens* genome. The Circo map is structured from the inner to the outer circle to display the distribution of the average GC content, the distribution of the average repetitive sequence content, the distribution of the average number of protein-coding genes, and the lengths of ten pseudo-chromosomes (with a sliding window size of 1 Mb, the numbering chr1 to chr10 represents the ten pseudo-chromosomes). (b) Heatmap of interaction frequency of genomic fragments of *O. rufescens.*

We used 146.08 Gb of Hi-C sequencing data to scaffold the 403 contigs of the draft genome, enhancing the assembly to the chromosomal level. This resulted in a chromosomal-level genome size of 877,902,837 bp, 80 scaffolds total, ten of which are pseudo-chromosomes. The longest pseudo-chromosome measured 163,837,246 bp, with an average length of 10,973,785 bp, and an N50 of 157,110,771 bp. The genome draft was used to construct ten pseudo-chromosomes, with an anchored rate of 92.31%, from 372 contigs. The lengths of the chromosomes range from 18,071,128 bp to 163,837,246 bp. Chromosome 2 had the highest number of mounted contigs, totaling 81, while chromosome 8 had the fewest, with 18 (refer to supplementary table S4, Supplementary Material online and Fig. 1a and b).

The BUSCO assessment results show that 98.4% of genes in the insecta_odb10 gene set were successfully detected, of which 96.8% are single-copy and 1.6% are multi-copy, with fragmented genes accounting for 0.8% (refer to supplementary fig. S4, Supplementary Material online). The BUSCO assessment results indicate that the final genome of *O. rufescens* at the chromosome level was highly complete.

### Genome Structure Annotation

Approximately 310 Mb of the *O. rufescens* genome was annotated as repetitive, constituting 35.3% of the entire genome. The highest portion of these repetitive sequences are Long Interspersed Nuclear Elements (LINEs), amounting to 21.6%. This is followed by DNA transposons, Short Interspersed Nuclear Elements (SINEs), Long Terminal Repeats (LTRs), and Penelope, which constitute 6.32%, 3.56%, 1.78%, and 0.33% of the genome, respectively (refer to supplementary table S5, Supplementary Material online).

The tRNAscan-SE predicted 15,481 tRNA genes, while RNAmmer identified 4,082 rRNA genes, comprising of 4,035 8S rRNA genes, 24 18S rRNA genes, and 23 28S rRNA genes. Additionally, Infernal software, using the Rfam database, predicted 22,655 noncoding genes, with a total length of 2,175,053 bp. The genome contains a variety of noncoding genes, including 5,637 rRNA genes, 634 snRNA genes, 74 miRNA genes, 15,806 tRNA genes, 3 lncRNA genes, 47 ribozymes, and 454 other types of noncoding genes (refer to Table 1 and supplementary table S6, Supplementary Material online).

A total of 15,031 protein-coding genes were annotated. The average gene length was 16,406 bp (refer to supplementary tables S7 and S8, Supplementary Material online). Further analysis of the gene structure revealed that the total length of cDNA sequences was 23.67 Mb, with the longest cDNA measuring 39,024 bp and an average length of 1,576 bp. The protein sequences have a total length of 7.88 million amino acids (Maa). The longest coding sequence is 13,007 amino acids (aa), with an average length of 524 aa (refer to supplementary table S8, Supplementary Material online).

The coding genes of *O. rufescens* comprise 15,031 genes, constituting a total of 106,993 exons. Collectively, these exons span 23.68 Mb, with an average length of 221 bp. A total of 91,980 introns are present, measuring 222.63 Mb in combined length and averaging 2,420 bp each. Notably, the length of introns exceeds that of exons. On average, each gene contains seven exons and six introns. The *O. rufescens* genome encompasses 15,093 intergenic regions. These intergenic regions span a total of 631.6 Mb, with an average length of 41,847 bp (refer to supplementary S8, Supplementary Material online).

Then conducted on the number of protein-coding genes located on the ten pseudo-chromosomes and other sequences, with chromosome 1 harboring the most genes, totaling 3,060 (refer to supplementary fig. S3a, Supplementary Material online). Separate statistics were collected on the length and proportion of exons, introns, and intergenic regions on the ten pseudo-chromosomes and other sequences (refer to supplementary fig. S3b, Supplementary Material online). The proportion of exons, introns, and intergenic regions is consistent across the ten pseudo-chromosomes, except for chromosome 10 which has a higher exon proportion of 4.21% compared to the average proportion of 2.73%. The remaining sequences have distinct features, with exons accounting for only 1.42% of the proportion (refer to supplementary fig. S3b, Supplementary Material online and supplementary table S9, Supplementary Material online).

The BUSCO assessment of protein-coding genes showed that 97.8% of the genes in the insecta_odb10 gene set were successfully detected, with 96.3% being single-copy, 1.6% multi-copy, and 0.5% being fragmented genes (refer to supplementary fig. S5, Supplementary Material online).

### Genome Function Annotation

The functions of the 15,031 protein-coding genes were determined by eight functional databases. The eggNOG database annotated 11,801 genes, the KEGG database annotated 7,139 genes, the Pfam database annotated 11,027 genes, the KOG database annotated 8,692 genes, the Swissprot database annotated 9,513 genes, the GO database annotated 9,397 genes, the NR database annotated 12,406 genes, and the TrEMBL database annotated 12,364 genes (refer to supplementary fig. S6, Supplementary Material online). In total, 12,823 genes were annotated by at least one database, and 5,769 genes were annotated by all eight databases. Additionally, we mapped the second- and third-generation transcriptome data to the genome data. The results showed that all mapping rates were above 96%, indicating the high quality of the genome annotation (refer to supplementary table S10, Supplementary Material online). Ylla et al. (2021) reported high-quality genome assemblies for *G. bimaculatus* and *N. kohalensis*, with the annotated protein-coding genes totaling 17,871 and 12,767, respectively. These figures are relatively close to our annotation results.

## Methods

### Sampling and DNA/RNA Extraction

This study collected a total of 30 females of *O. rufescens* for research. The samples were collected in 2021 within the campus of Shaanxi Normal University. All specimens were kept alive and then transferred to the lab for further processing. The QIAGEN DNeasy Blood & Tissue Kit method was used for DNA extraction, and the TRIzol method was used for RNA extraction.

### Library Construction and Genome Sequencing

Five sequencing libraries were constructed, divided into two categories. The second-generation libraries included genome, Hi-C, and transcriptome libraries, all sequenced on the Illumina Novaseq 6,000 platform using PE150 mode and a 350 bp insert size. The genome library involved DNA fragmentation, end repair, adapter ligation, purification, and PCR amplification. The Hi-C library involved fixation, enzyme digestion, biotin labeling, protein digestion, sonication, biotin capture, adapter ligation, and PCR amplification. The transcriptome library involved reverse transcription of mRNA to cDNA, followed by fragmentation and repair. The third-generation libraries included genome and transcriptome types, both sequenced on the PacBio Sequel IIe platform in HiFi mode. The genome library involved DNA extraction, enzyme digestion, end repair, adapter ligation, and purification. The transcriptome library involved RNA extraction, end repair, adapter ligation, and purification.

### Preprocessing and Genome Size Estimation

We analyzed raw sequencing data from second- and third-generation libraries using Seqkit v2.5.0 (Shen et al. 2016) and assessed exogenous DNA contamination through Blast v2.12.1 (Altschul et al. 1990) alignment with the NCBI Nucleotide Database. Quality of second-generation data was evaluated using Fastqc v0.11.9 (Wingett and Andrews 2018).

For the genome size estimation, we employed the sub-command “kmerfreq” from the GCE v1.0.2 (Liu et al. 2013) to perform. Frequencies of 17-mers were generated based on high-quality PE reads. The genome size was calculated using the formula *G* = *N*_k-mer_/Daverage_k-mer_, where *N*_k-mer_ represents the total number of k-mers, and Daverage_k-mer_ the average depth (Guo et al. 2015). The genome size was also determined via flow cytometry, employing *Periplaneta americana* male (1C = 3.41 pg) as the internal standard. This process adhered to the protocols established by Mao et al. (2020) and Gregory and Johnston (2008). The genome size was calculated using the formula: Genome size_sample_ = Genome size_internal standard_ × (sample 2C mean peak position/internal standard 2C mean peak position) (Lower et al. 2017).

### Genome Assembly

The preliminary genome assembly of *O. rufescens* was conducted using Hifiasm v0.16.1 (Cheng et al. 2021). Redundancies were removed post-assembly using Purge_dups v1.2.6 (https://github.com/dfguan/purge_dups) and second-generation library data to produce a draft genome.

The draft genome of *O. rufescens* was further scaffolded using high-quality data from the Hi-C library with YaHS v1.2a.1 (Zhou et al. 2023). Prior to scaffolding, the high-quality Hi-C library data were aligned to the genome draft using BWA v0.7.17 (Li and Durbin 2010) and Samtools v1.14 (Danecek et al. 2021). After scaffolding, manual adjustments were made using Juicebox v2.15 (Durand et al. 2016) to correct misassemblies, translocations, and inversions, as well as to optimize chromosomal boundaries. The assembly quality was assessed using BUSCO v5.2.2 (Manni et al. 2021) with the arthropoda_odb10 and insecta_odb10 single-copy ortholog datasets.

### Repeat Annotation, Gene Prediction, and Function Annotation

De novo repeat sequences in the *O. rufescens* genome were predicted using RepeatModeler v2.0.1 (Flynn et al. 2020), and integrated with sequences from the RepBase (Bao et al. 2015) and Dfam databases (Storer et al. 2021). RepeatMasker v4.1.4 (Tarailo-Graovac and Chen 2009) was then used for annotating and masking these repeats. For RNA genes, RNAmmer v1.2 (Lagesen et al. 2007) annotated rRNA genes, while tRNAscan-SE v2.0 annotated tRNA genes. Other ncRNAs were annotated using Infernal v1.1.4 (Nawrocki and Eddy 2013), with selection based on E-values and overlap values.

Three strategies were employed for predicting protein-coding genes: Transcriptome-based Prediction: HiFi reads from third-generation transcriptomics were processed using Smrtlink to obtain full-length Isoform sequences, trim polyA and multimer sequences, cluster Isoform sequences, and align them to the reference genome. For second-generation transcriptomics data, Hisat v2.2.1 (Kim et al. 2015) aligned the data to the genome, integrated across three samples using Samtools, and assembled using Stringtie v2.2.1 (Pertea et al. 2015). Coding regions were predicted using TransDecoder v5.5.0 (https://github.com/TransDecoder/TransDecoder). Homology-based Prediction: Homologous genes were predicted using Exonerate v2.4.0 (Slater and Birney 2005) comparing to species including *Drosophila melanogaster*, *T. oceanicus*, *Xya riparia*, *Locusta migratoria*, and *G. bimaculatus*. De novo Structure-based Prediction: Gene structures were predicted using Braker3, combining second-generation transcriptomic data and homologous protein data from related species.

EggNOG (Evolutionary Genealogy Of Genes: Non-supervised Orthologous Groups, http://eggnog6.embl.de/), KEGG (Kyoto Encyclopedia of Genes And Genomes, https://www.kegg.jp/), Pfam (https://rfam.org/), KOG (Clusters Of Eukaryotic Orthologous Groups, https://ftp.ncbi.nih.gov/pub/COG/KOG/), Swissprot (https://www.uniprot.org/), GO (Gene Ontology, http://geneontology.org/), NR (Non-Redundant Protein Sequence Database, https://ftp.ncbi.nlm.nih.gov/blast/db/FASTA/nr,gz), and TrEMBL (https://www.uniprot.org/) databases were used for alignment and to functionally annotate the predicted genes. Finally, Bowtie v1.2.2 and minimap v2.24-r1122 were used to map second- and third-generation RNA reads to the genome data, respectively, to validate the completeness of the genome annotation.

## Supplementary Material

evae145_Supplementary_Data

## Data Availability

We have uploaded the raw sequencing data (including second-generation, third-generation, and Hi-C data) to the NCBI database. The BioProject accession number is PRJNA1097282, and BioSample accession number is SAMN40864211. The data of genome assembly and annotations are available on figshare, and can be accessed at https://doi.org/10.6084/m9.figshare.25573647.v1.
