# Supplementary material for: A Chromosome-Level Genome Assembly and Annotation for the Oecanthus rufescens (Orthoptera: Oecanthidae)
Source: Genome Biol Evol. 2024 Jul 1;16(7):evae145. doi: 10.1093/gbe/evae145 (PMC11243396; doi:10.1093/gbe/evae145)
Supplement: evae145_Supplementary_Data [file evae145_supplementary_data.zip › Suppementary Figures.docx]

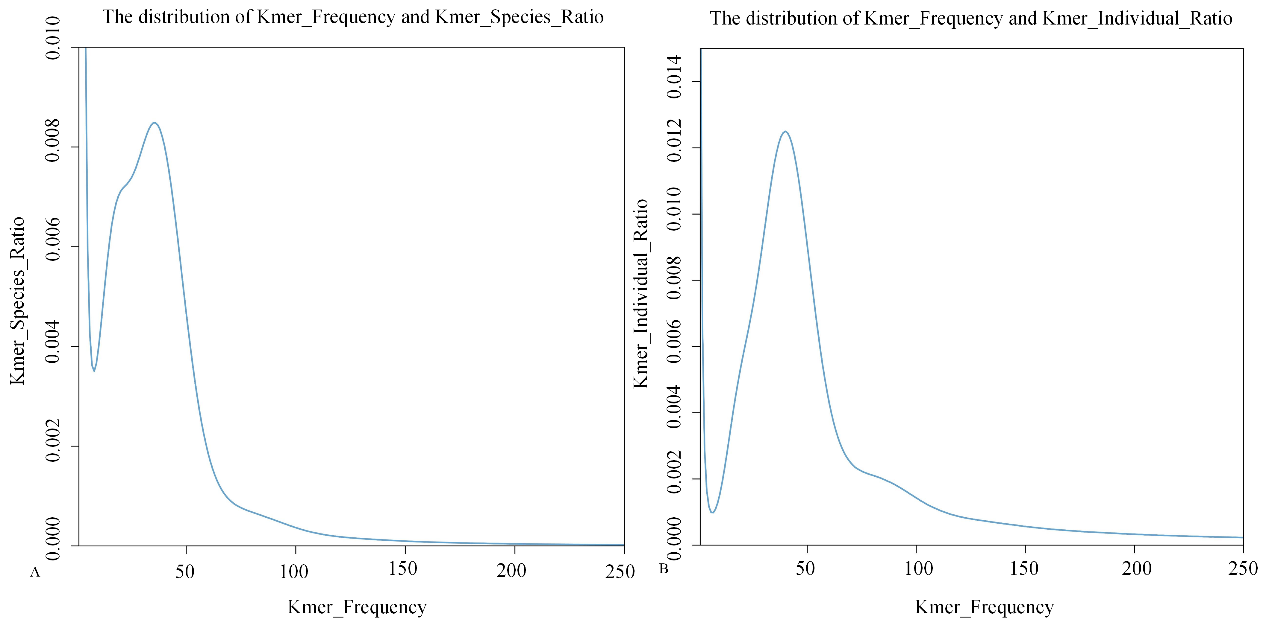


**Fig. S1.** Distribution diagram of Kmer frequency. (A) The horizontal axis represents Kmer frequencies; the vertical axis represents Kmer species ratios. (B) The horizontal axis represents Kmer frequency; the vertical axis represents Kmer number ratio.


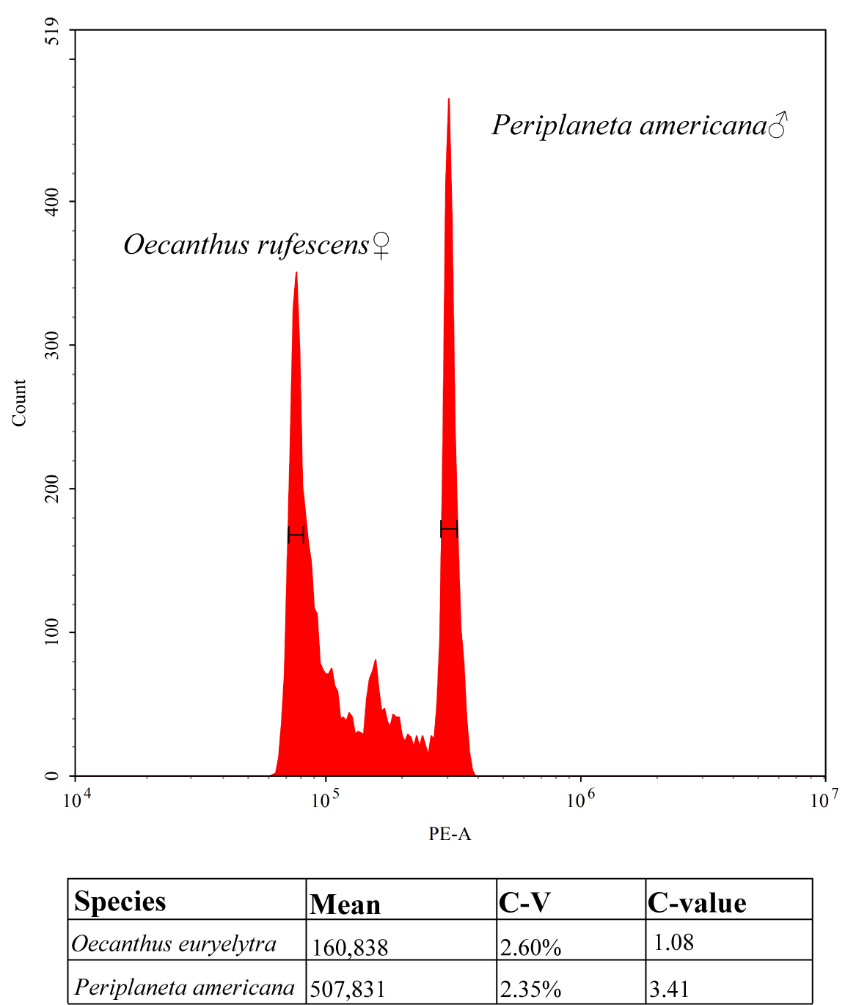


**Fig. S2.** Estimating genome size of *O. rufescens* using flow cytometry.


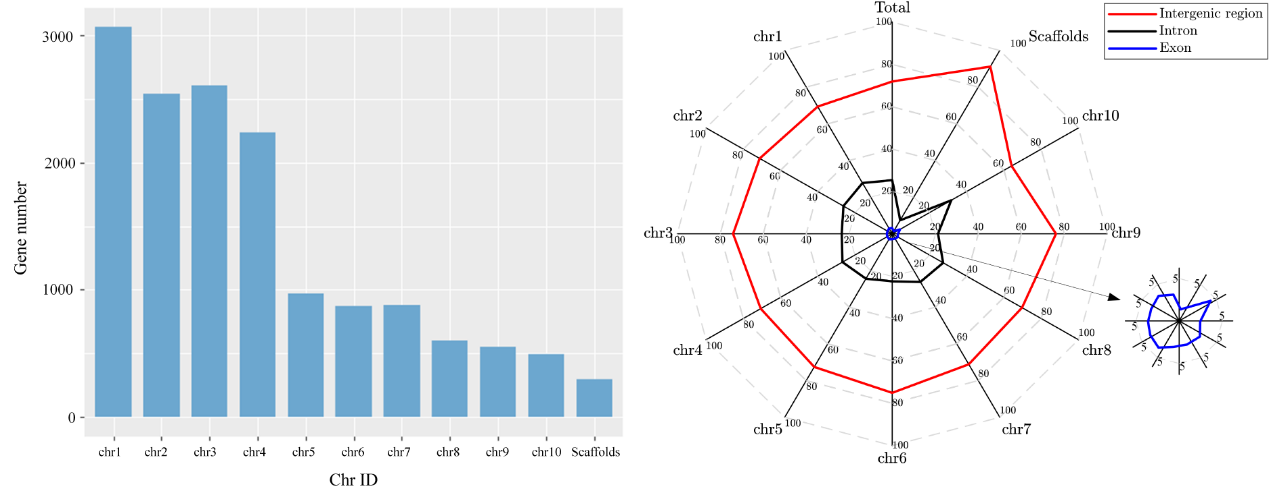


**Fig. S3.** (A) Statistical chart of protein-coding gene count of *O. rufescens* genome. (B) Radar map of genetic characters of *O. rufescens* genome.


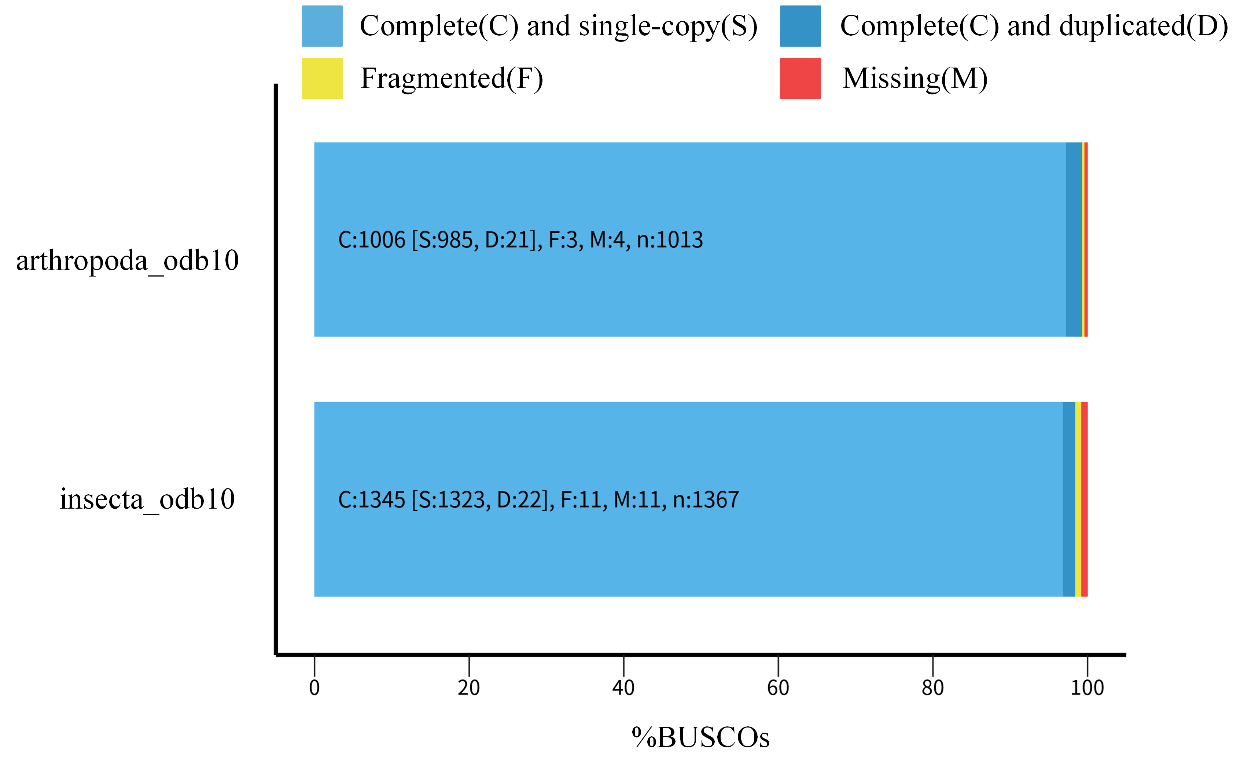


**Fig. S4.** BUSCO Estimation result of *O. rufescens* genome.


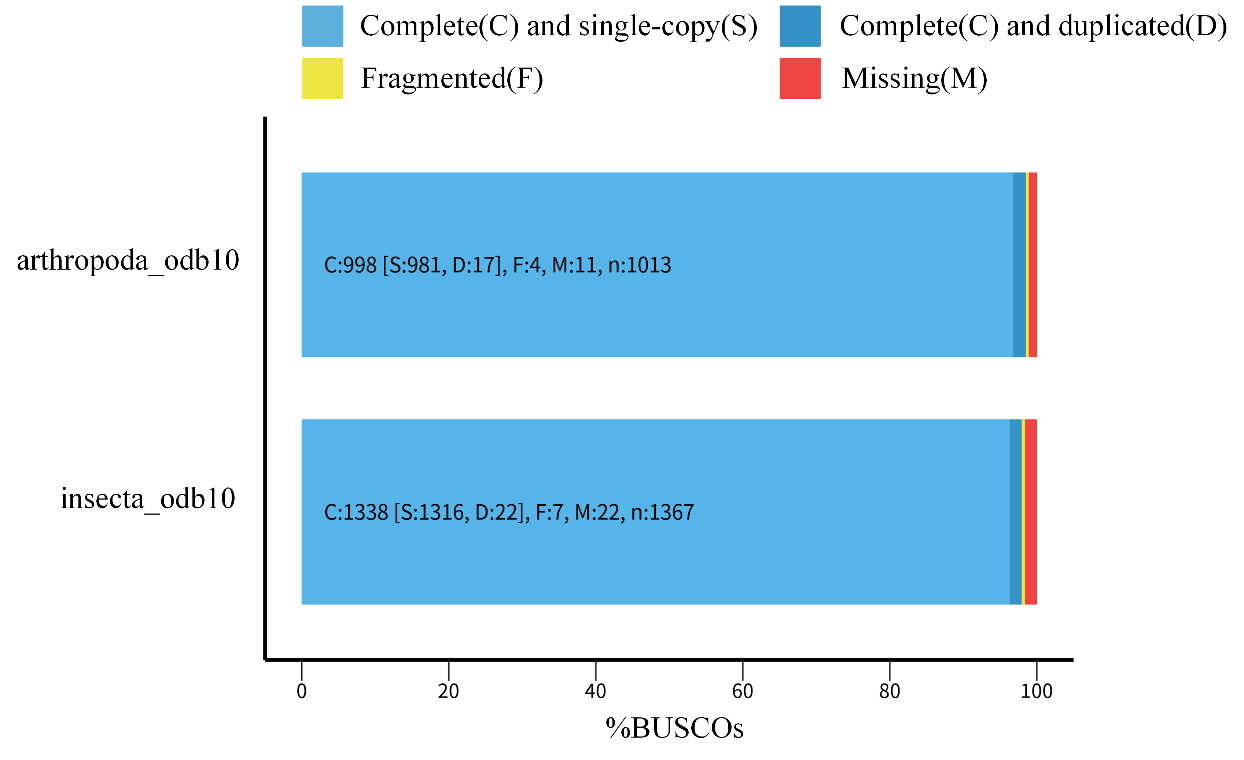


**Fig. S5.** BUSCO Estimation result of *O. rufescens* protein-coding genes.


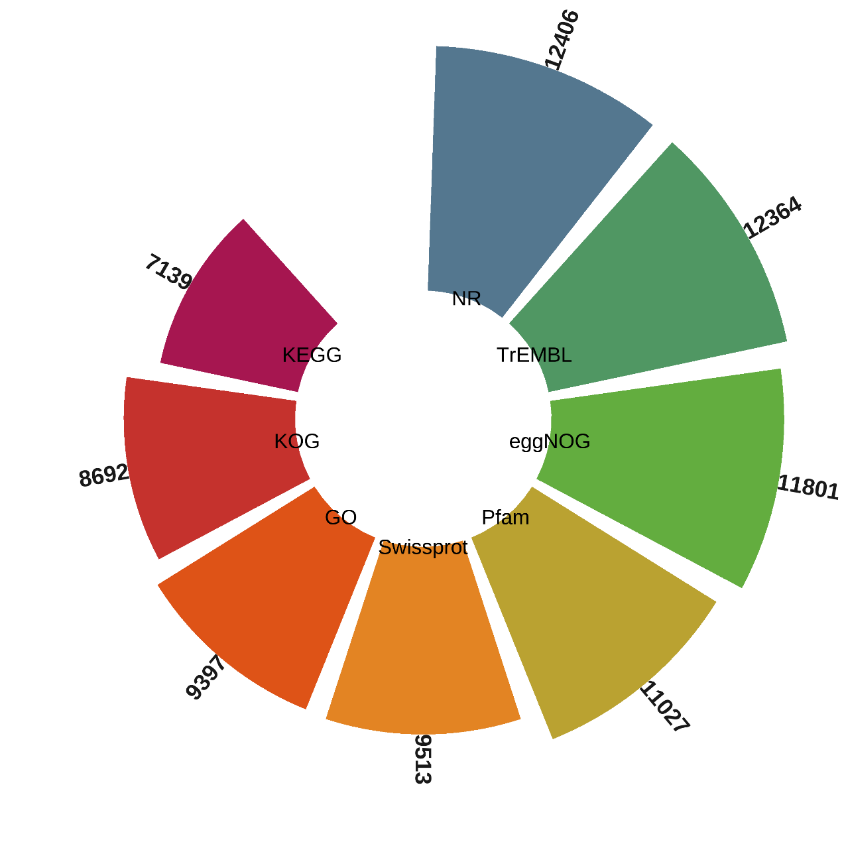


**Fig. S6.** Chart of annotation results of eight function databases of protein-coding gene of *O. rufescens.*
